# Genotype Distribution and Sequence Variation of Hepatitis E Virus, Hong Kong

**DOI:** 10.3201/eid1505.081579

**Published:** 2009-05

**Authors:** Wai-Yip Lam, Rickjason C.W. Chan, Joseph J.Y. Sung, Paul K.S. Chan

**Affiliations:** The Chinese University of Hong Kong, Shatin, New Territories, Hong Kong Special Administrative Region, People’s Republic of China

**Keywords:** Zoonoses, viruses, hepatitis E virus, HEV, epidemiology, genotype, Hong Kong, China, dispatch

## Abstract

Most acute cases of infection with hepatitis E virus (HEV) in Hong Kong were autochthonous, sporadic, and occurred in older adults. All except 1 isolate belonged to genotype 4; most were phylogenetically related to swine isolates. The epidemiology is similar to that in industrialized countries, where zoonosis is the major source of HEV infection in humans.

Hepatitis E virus (HEV) is a major cause of enterically transmitted acute hepatitis throughout Asia, the Middle East, and Africa. Large outbreaks resulting from fecal contamination of drinking water are confined mainly to developing countries (*1*), whereas sporadic cases in industrialized countries are thought to be zoonotic, with swine being the most likely reservoir (*2*). Studies in Hong Kong reported a seroprevalence of 16%–19% (*3*). No large outbreak has been recorded in Hong Kong, and the sporadic cases are believed to be imported (*4*). Our study examined the epidemiology and genotype distribution of HEV infections in Hong Kong in an effort to improve control of this disease.

## The Study

We studied patients admitted to the Prince of Wales Hospital, Hong Kong Special Administrative Region, who had laboratory-confirmed acute hepatitis E during 2002– 2007. Acute HEV infection was diagnosed on the basis of clinical manifestations of acute hepatitis, elevation of hepatic parenchymal enzyme levels, and presence of anti-HEV immunoglobulin (Ig) M as determined by an HEV IgM kit (Biotec Laboratories Ltd., Suffolk, UK). Clinical data were retrieved from patients’ records in the computerized clinical management system of the hospital. HEV RNA was then amplified from patients’ stored serum by using a nested reverse transcription–PCR (RT-PCR) specific for open reading frame (ORF) 2 with external primers ConsORF2-s1 (5′-GACAGAATTRATTTCGTCGGCTGG-3′) and ConsORF2-a1 (5′-CTTGTTC RTGYTGGTTRTCATAATC-3′) and internal primers ConsORF2-s2 (5′-GTYGTCTCRGCCAATGGCGAGC-3′) and ConsORF2-a2 (5′-GTTCRTGYTGGTTRTCATAATCCTG-3′) (*5*). Positive samples were subjected to another nested RT-PCR targeting ORF1 with external primers HE5-1 (5′-TCGATGCCATGGAGGCCC-3′) and HEVORF1-1as (5′-GGCCATTGCCTCCGCAACATC-3′) and internal primers HE5-2 (5′- GCCYTKGCGAATGCTGTGG-3′) and HEVORF1-2as (5′- ACCATCAAAGCAGTAAGTCCG-3′) (*6*). The ORF2 (145-bp) and ORF1 (364-bp) PCR products were sequenced, and sequence fragments were aligned by CLUSTALX 2.0 (*7*). Phylogenetic trees were constructed by using the neighbor-joining method (*8*), and rooted phylogenetic trees were generated by using PAUP* version 4.0b (*9*). Bootstrapping values obtained from 1,000 resamplings of the data were performed to assess the robustness of trees (*10*). The final tree was obtained with the FigTree program, version1.1.2 (*11*). Nucleotide sequence similarity among isolates was analyzed by using the Bioedit software (*12*). The sequence data were deposited in GenBank under accession nos. FJ438395–FJ438427 and FJ438428–FJ438460, respectively.

We identified 57 patients, of whom 56 were Chinese. All patients were negative for acute hepatitis A and B markers. Nineteen were females (none pregnant) 14–82 years of age (median age 57 years, interquartile range [IQR] 43–74 years); 38 were men 26–76 years of age (median age 52 years, IQR 38–67 years). No significant difference in age was observed (p = 0.323 by Mann-Whitney U test). Eleven patients were hepatitis B carriers, 1 had cirrhosis, and 1 was a hepatitis C carrier.

Forty-eight (84%) patients had no history of travel during the prior 6 months. Nine (16%) had traveled outside Hong Kong (6 to People’s Republic of China, 1 to Macau, 1 to South Korea, 1 to the United States) 1–4 weeks before illness onset. All cases were sporadic.

The highest liver function levels recorded for the women were serum alanine aminotransferase (ALT) 261–6,500 IU/L (median 1,280 IU/L, IQR 434–8,322 IU/L, reference <58 IU/L). Serum alkaline phosphatase (ALP) levels ranged from 111 to 469 IU/L (median 214 IU/L, IQR 178–268 IU/L, reference 45–145 IU/L), and total serum bilirubin levels ranged from 10 to 565 μmol/L (median 85 μmol/L, IQR 30–146 μmol/L, reference, <15 μmol/L). The highest liver function levels recorded for the men were serum ALT 253–4,525 IU/L (median 1,714 IU/L, IQR 1,043–2,608 IU/L, reference <58 IU/L), serum ALP 76–912 IU/L (median 188 IU/L, IQR 132–261 IU/L, reference 35–100 IU/L), and total serum bilirubin 10–544 μmol/L (median 112 μmol/L, IQR 72–227 μmol/L, reference <15 μmol/L). No fulminant hepatitis was recorded, and all patients recovered.

Thirteen patients had other medical conditions including end-stage renal failure, diabetes, ischemic heart disease, colon cancer, system lupus erythematous, thyroitoxicosis, hepatitis B liver cirrhosis, and previous liver transplantation. Patients with a medical condition had significantly higher levels of serum ALP (median 267 IU/L, IQR 86–1,703 IU/L) than did those without medical conditions (median 192 IU/L, IQR 131–164 IU/L; p = 0.03 by Mann-Whitney U test).

Phylogenetic analyses of the ORF2 fragments from 46 patients and ORF1 fragments from 33 patients showed complete agreement (Figure), with most (45 [98%]) belonging to genotype 4. The remaining isolate was genotype 3 (HK14) obtained from a woman who had no history of travel. Most of the Hong Kong isolates clustered closely with a swine isolate reported from Guangxi Province, China (accession no. EU676172). Furthermore, the ORF2 phylogenetic tree showed our isolates were closely related to those reported recently from Beijing, China (accession nos. EU107400–EU107474) (*13*).

**Figure:**
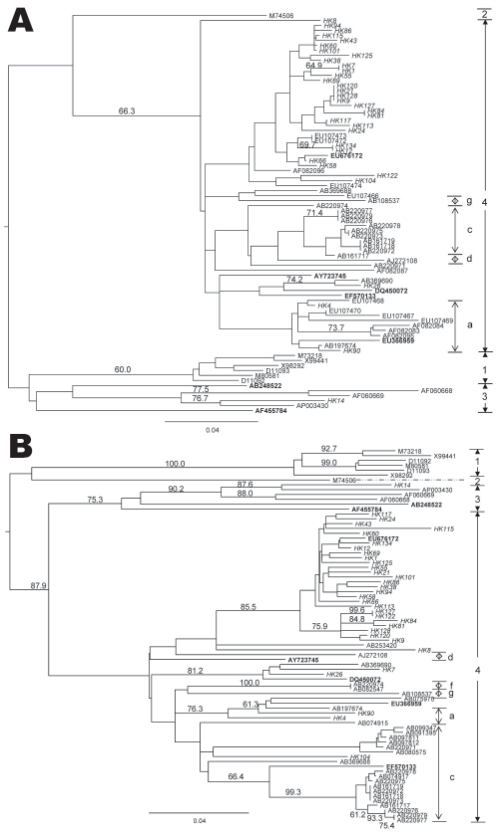
Phylogenetic tree showing the relationship of hepatitis E virus (HEV) isolates from Hong Kong. Trees were constructed by the neighbor-joining method based on the partial nucleotide sequence of the open reading frame (ORF) 2 (A) and ORF1 (B) regions of HEV samples. Genotypes are indicated by numbers and subtypes by letters on the right. Branch lengths are proportional to genetic distance. Scale bars indicate 0.04 nt substitutions per position. Bootstrap values for the various branches are shown as percentages of trees obtained from 1,000 resamplings of the data. Sequences used for phylogenetic analysis were isolates from genotype 1: Burma (GenBank accession nos. M73218; D10330), Pakistan (M80581), India (X98292; X99441), and China (D11092; D11093); genotype 2: Mexico (M74506); genotype 3: USA (AF060668; AF060669), Japan (AP003430); and genotype 4: China (AB108537; AB197674; AF082083; AF082084; AF082087; AF082095; AF082096; AJ272108; EU107466-74), Japan (AB074915; AB074917; AB080547; AB091395; AB097812; AB099347; AB161717-19; AB220971-79; AB253420; AB369688; AB369690). Branches of swine HEV genotype 3 sequences (AB238522; AF455784 [experimentally infected swine]) and swine HEV genotype 4 sequences (AY723745; DQ450072; EF570133; EU366959; EU676172) are included in the analysis. Accession numbers in **boldface** are swine isolates. All isolates from the current study have a prefix HK followed by a number in italics.

We further analyzed the sequence variation of 32 HEV genotype 4 isolates for which ORF1 and ORF2 sequences were generated in the current study. The nucleotide sequence similarity was 79.9% for the ORF1 region (nt 170–448), and 86.4% for ORF2 (nt 6409–6504). For comparison, the sequence similarity for HEV isolates collected elsewhere that had been deposited in GenBank was 73.8% for ORF1 (nt 170–448) and 74.0% for ORF2 (nt 6409–6504). Regions within nt 171–221, 280–310, and 6,461–6,495 were most conserved and represented the best targets for primer or probe design.

## Conclusions

Our study showed that most HEV cases in Hong Kong were sporadic and autochthonous. Although a substantial proportion (21%) of patients were hepatitis B or C carriers, all diseases had a self-limiting course. This finding could be related to the circulation of relatively mild genotypes 3 and 4 in this locality. The epidemiology of HEV in Hong Kong resembled industrialized countries with a predilection for older adults, rather than older children and young adults as occurs in developing countries. Patients with HEV infections were older than those with hepatitis A, which peaked in persons 20–29 years of age, as reported by Chau et al. (*14*).

The distribution of HEV genotypes is related to geographic location and the mode of spread (*2*). Genotype 1 is epidemic in developing countries in Asia and North Africa. Genotype 2 is found in Mexico and in central African countries. Genotypes 1 and 2 occur only in humans. Genotype 3 is widely distributed and has been isolated from humans in North and South America, Europe, Japan, and the Pacific region and in domestic pigs in many countries except in Africa. Genotype 4 has been isolated from humans in China, Japan, Taiwan, and Vietnam and from domestic pigs, boar, and deer in many countries (*2**,**5**,**6*). Genotype 4, the predominant genotype in Hong Kong, is less virulent; it is responsible for occasional cases of clinical hepatitis in industrialized countries. A recent study from Guangzhou in southern China showed that most (39/41) HEV isolates found there were similar to Burmese-like isolates (genotype 1) (*15*). Although Hong Kong is near Guangzhou, none of our isolates were genotype 1. This finding could imply that the poor hygienic conditions required for sustaining the circulation of genotypes 1 and 2 do not exist in Hong Kong.

HEV infections in Hong Kong are mainly acquired locally. The sporadic nature, older age of affected patients, and predominance of genotype 4 correspond with the epidemiology in industrialized countries where zoonosis is the major source of infection. Public health control should focus on zoonotic, especially swine, foodborne transmission as a source of human HEV infection in Hong Kong.

## References

[1] Panda SK, Thakral D, Rehman S. Hepatitis E virus. Rev Med Virol. 2007;17:151–80. 10.1002/rmv.522 17051624

[2] Purcell RH, Emerson SU. Hepatitis E: an emerging awareness of an old disease. J Hepatol. 2008;48:494–503. 10.1016/j.jhep.2007.12.008 18192058

[3] Wong KH, Liu YM, Ng PS, Young BW, Lee SS. Epidemiology of hepatitis A and hepatitis E infection and their determinants in adult Chinese community in Hong Kong. J Med Virol. 2004;72:538–44. 10.1002/jmv.20040 14981755

[4] Lai JY. Hepatitis A and E in Hong Kong. Hong Kong Med J. 1997;3:79–82. 11847359

[5] Wang Y, Ling R, Erker JC, Zhang H, Li H, Desai S, A divergent genotype of hepatitis E virus in Chinese patients with acute hepatitis. J Gen Virol. 1999;80:169–77. 9934699 10.1099/0022-1317-80-1-169

[6] Hijikata M, Hayashi S, Trinh NT, Ha le D, Ohara H, Shimizu YK, et al. Genotyping of hepatitis E virus from Vietnam. Intervirology. 2002;45:101–4. 10.1159/000063231 12145542

[7] Larkin MA, Blackshields G, Brown NP, Chenna R, McGettigan PA, McWilliam H, Clustal W and Clustal X version 2.0. Bioinformatics. 2007;23:2947–8. 10.1093/bioinformatics/btm404 17846036

[8] Saitou N, Nei M. The neighbor-joining method: a new method for reconstructing phylogenetic trees. Mol Biol Evol. 1987;4:406–25. 3447015 10.1093/oxfordjournals.molbev.a040454

[9] Swofford DL. 2003. PAUP*. Phylogenetic analysis using parsimony (*and other methods). Version 4. Sunderland (MA): Sinauer Associates [cited 2009 Feb 9]. Available from http://paup.csit.fsu.edu/win.html

[10] Felsenstein J. Confidence limits on phylogenies: an approach using the bootstrap. Evolution Int J Org Evolution. 1985;39:783–91. 10.2307/2408678 28561359

[11] Rambaut A. 2008. FigTree v1.1.2 [cited 2009 Feb 9]. Available from http://tree.bio.ed.ac.uk /software/figtree

[12] Hall TA. BioEdit: a user-friendly biological sequence alignment editor and analysis program for Windows 95/98/NT. Nucleic Acids Symp Ser. 1999;41:95–8.

[13] Zhao C, Li Z, Yan B, Harrison TJ, Guo X, Zhang F, Comparison of real-time fluorescent RT-PCR and conventional RT-PCR for the detection of hepatitis E virus genotypes prevalent in China. J Med Virol. 2007;79:1966–73. 10.1002/jmv.21040 17935186

[14] Chau TN, Lai ST, Lai JY, Yuen H. Acute viral hepatitis in Hong Kong: a study of recent incidences. Hong Kong Med J. 1997;3:261–6. 11847370

[15] Wei S, Xu Y, Wang M, To SS. Phylogenetic analysis of hepatitis E virus isolates in southern China (1994–1998). J Clin Virol. 2006;36:103–10. 10.1016/j.jcv.2006.03.001 16621689

